# TMBIM6-mediated miR-181a expression regulates breast cancer cell migration and invasion via the MAPK/ERK signaling pathway

**DOI:** 10.7150/jca.81600

**Published:** 2023-02-22

**Authors:** Yeokyung Shin, Hye Yeon Choi, Yeonjoo Kwak, Gwang-Mo Yang, Yeojin Jeong, Tak-Il Jeon, Jaekwon Seok, Eung-Ryoung Lee, Jung-Hyun Kim, Kilsoo Jeon, Ahmed Abdal Dayem, Kyung Min Lim, Ssang-Goo Cho

**Affiliations:** 1 Department of Stem Cell & Regenerative Biotechnology and Institute of Advanced Regenerative Science, Konkuk University, 120 Neungdong-ro, Gwangjin-gu, Seoul 05029, Republic of Korea.; 2 R&D Team, StemExOne co., ltd. 303, Life Science Bldg, 120, Neungdong-ro, Gwangjin-gu, Seoul 05029, Republic of Korea

**Keywords:** TMBIM6, miRNA-181a, Breast cancer, MAPK/ ERK signaling pathway, Snail, FOSL-1/c-Jun

## Abstract

Transmembrane Bax Inhibitor Motif-containing 6 (TMBIM6) has been reported to regulate cell death pathways and is overexpressed in several types of cancers. In this study, we investigated whether high expression of TMBIM6 in breast cancer was significantly associated with cancer invasiveness. Knockdown of *TMBIM6* reduced proliferation and migration of invasive breast cancer cells through downregulation of the MAPK/ERK signaling pathway. Moreover, we suggested that expression of miR-181a was significantly suppressed upon *TMBIM6* knockdown. In contrast, overexpression of *TMBIM6* significantly increased cell invasion and migration through up-regulation of mesenchymal markers and matrix metalloproteinase-9 (MMP-9) and enhanced activation of the MAPK/ERK signaling pathway. We also observed that up-regulation of TMBIM6 significantly increased the expression of miR-181a by TMBIM6-mediated pathway. TMBIM6 and miR-181a-mediated ERK activation induced the expression of Snail-1 and Snail-2 in FOSL-1/C-JUN-dependent manner. Overall, our data demonstrated that TMBIM6-induced miR-181a up-regulation plays an important role in the efficient modulation of migration and invasion of breast cancer cells.

## Introduction

Breast cancer is the second most common cause of cancer-related death and is the most frequently diagnosed cancer in women 1, 2. Several studies so far have shown that abnormal apoptotic signaling pathways are a major cause of the initiation and progression of this disease 3. However, despite many studies of breast cancer progression, the factors modulating proliferation in breast cancer cells are complex and not fully defined.

Known as an anti-apoptotic membrane protein, Transmembrane Bax Inhibitor motif-containing 6 [TMBIM6 or Bax inhibitor-1 (BI-1)] is a type of regulator for cell death pathways. TMBIM6 interacts with Bcl-2 and Bcl-XL, but not Bax or Bad, and overexpression of TMBIM6 in mammalian cells results in suppression of apoptosis induced by diverse apoptotic stimuli 4, 5. TMBIM6 is overexpressed in several tumor types, where it has important roles in tumor progression and malignancy 6, 7. Furthermore, specific downregulation of TMBIM6 by RNA interference resulted in death in human prostate and breast cancer cells 8, 9. Several clinical studies have indicated an association between TMBIM6 expression and breast cancer 9-11. Previously, we have shown that MAPK/ERK activation by TMBIM6 overexpression has an important role in its anti-apoptotic effect in diverse cell lines, which is closely related to its anti-inflammatory effect or function in stem cell differentiation 12. However, despite evidence of TMBIM6 overexpression in cancer cells, the molecular mechanisms by which TMBIM6 regulates cancer cell phenotypes are not clear.

Epithelial to mesenchymal transition (EMT), a process through which cells revert from an epithelial to mesenchymal state is characterized by reduced E-cadherin expression and adhesion of cells, leading to increased cell migration 13. Loss of E-cadherin, a protein important for cell-cell adhesion, results in a decrease in cytokeratin levels and increased expression of mesenchymal markers such as N-cadherin, Vimentin, and matrix metalloproteinases (MMPs) 14, 15. This process is generally initiated in aggressive cancer cells and is essential for cancer cell invasion into surrounding tissues. Moreover, MMPs enable cell migration, invasion, and metastasis in cancer 16, 17. The ability of MMPs to degrade components of the extracellular matrix has led to extensive characterization of their cellular functions under physiological and pathological conditions 18.

Several distinct MAPKs, including the p42/p44 extracellular signal-related kinases (ERK1/2), c-Jun N-terminal protein kinase (JNK)/stress-activated protein kinase, and p38 MAPK are involved in various cellular functions, including proliferation, apoptosis, differentiation, cytoskeleton remodeling, and cell cycle regulation 19, 20. Furthermore, various evidence suggests a role for MAPK family members in cell motility and invasion 21, 22. The ERK pathway in particular has been reported to be involved in migration and invasion of human breast and prostate cancer cells 23-25.

MicroRNAs (miRNAs) have been recognized as regulatory RNAs with significant roles in multiple pathological processes 26, 27. miRNAs play crucial roles in cell proliferation, apoptosis and tumorigenesis, and their aberrant expression is thought to be relevant to the cancer progression 28, 29. For example, it has been shown that overexpression or inhibition of miRNA in breast cancer is associated with the development and progression of breast cancer 30, 31. Especially, several studies have reported that miR-181a is up-regulated in various cases of cancer containing breast cancer and is related to chemoresistance 32-34.

To date, there is no report that has revealed the interaction between TMBIM6 and miR-181a in human cancers. In this study, we propose that TMBIM6-mediated miR-181a leads to migration and proliferation of breast cancer cells through activation of MAPK/ERK, which may affect malignant progression.

## Material and Methods

### Cell lines and DNA transfection

Human breast cancer MCF7, MDA-MB231, and MDA-MB468 cells were grown in DMEM (Sigma-Aldrich, Saint Louis, MO, USA) containing 10% fetal bovine serum (FBS, Hyclone Laboratories, Logan, UT, USA) and 100 U/mL penicillin-streptomycin (1% P/S; Thermo Fisher Scientific, Waltham, MA, USA). MCF10A cells, spontaneously immortalized human breast epithelial cell lines 35, were cultured in DMEM/F12 medium (Thermo Fisher Scientific) supplemented with 5% horse serum (Thermo Fisher Scientific), 0.5μg/ml hydrocortisone (Sigma-Aldrich), 10μg/ml insulin (Thermo Fisher Scientific), 20 ng/ml epidermal growth factor (EGF; Sigma-Aldrich), 0.1μg/ml cholera enterotoxin (Sigma-Aldrich), 1% P/S, 2 mM l-glutamine (Sigma-Aldrich), and 0.5μg/ml amphotericin B (Sigma-Aldrich). For DNA transfection, MCF10A cells were incubated overnight at a density of 3 × 10^6^ cells per 100 mm in a culture dish and transfected with the indicated expression vectors using Lipofectamine reagent (Thermo Fisher Scientific) according to the manufacturer's instructions. Validated human ERK siRNA and scrambled siRNA were obtained from Santa Cruz Biotechnology and PD98059 was taken from BioMol (Plymouth Meeting, PA, USA).

### Plasmid construction

pEF-HA-human *TMBIM6* or *TMBIM6-ΔC* plasmid was constructed as described previously 12. The human *TMBIM6* gene was amplified by PCR from human fetal brain cDNA (BD Biosciences, Franklin Lakes, NJ, USA) using the TMBIM6 primers: 5′-GGGAAGAATTCATGAACATATTTGATCGA-3′ (forward) and 5′-GGGAACTCGAGTCATTTCTTCTCTTTCTT-3′ (reverse). A C-terminal deletion mutant form of *TMBIM6* (*TMBIM6-ΔC*) was generated using PCR-based methods to delete the last nine C-terminal amino acids of the TMBIM6 protein using the C-terminal truncation primer: 5′-GGGAAGAATTCATGAACATATTTGATCGA-3′ and 5′-GGGAACTCGAGTCACTAGGACCGGTACTTA-3′. DNA fragments containing *TMBIM6* or *TMBIM6-ΔC* were subcloned into the BamHI/NotI (Takara Bio, Otsu, Japan) sites of the pEF-HA mammalian expression vector. Lentiviral *TMBIM6* shRNA plasmid was constructed as described before 36. A double-stranded oligonucleotide was designed according to a common sequence of *TMBIM6* sequence, as previously described 8, to allow the formation of a hair-pin structure in the expressed oligo-RNA; this oligonucleotide was cloned into the Lentiviral vector.

### Lentiviral particle production and transduction of target cells

HEK293T cells were co-transfected with the lentiviral packaging constructs and TMBIM6 shRNA plasmid. The virus-containing supernatants were harvested 72 h after transfection and filtered through a 0.45μM filter. Transduction efficiency was greater than 90% for all conditions as determined by GFP fluorescence.

### RT-PCR and quantitative real-time RT-PCR analyses

Total RNA was isolated from cells using Trizol reagent (Thermo Fisher Scientific) and RT-PCR analysis was performed using AMV-RT (Promega, Madison, WI, USA) according to the manufacturer's instructions. All samples were run in triplicate. The specific gene sequences are listed in Table 1. Quantitative real-time RT-PCR was performed with Fast SYBR Green Master Mix (Thermo Fisher Scientific) and relative gene expression was determined by normalizing to that of GAPDH using the comparative CT method.

### Immunofluorescence analysis

Immunofluorescent labeling on cover glass was performed as described previously 36. Briefly, cells were fixed with 4% cold paraformaldehyde for 10 min, followed by incubation in 4% normal goat serum (Vector Laboratories, Burlingame, CA, USA) and 1% bovine serum album (BSA) for 20 min for blocking. Cells were incubated with primary rabbit anti-N-cadherin or anti-MMP-9 antibodies overnight at 4°C. Sections were then incubated for 1 h with donkey anti-rabbit secondary antibodies (1:200) conjugated to fluorescein isothiocyanate (FITC) or tetramethylrhodamine isothiocyanate (TRITC; Thermo Fisher Scientific). For double-labeling, the cells were treated with DAPI (4'-6-diamidino-2-phenylindole; Vector Laboratories) or with TO-PRO-3 (1:200; Molecular Probes, Eugene, OR, USA) overnight at 4°C. Immunofluorescent images were analyzed using confocal microscopy (Nikon C1 laser scanning confocal microscope).

### Western blot analysis

SDS-PAGE and Western blotting were conducted as described previously 12, 37. Cells in 100 mm dishes were washed 3 times in ice-cold PBS, scraped from the dishes, and collected in extraction buffer [1% Triton X-100 (Thermo Fisher Scientific), 100 mM Tris-HCl (Sigma-Aldrich), pH 7.5, 10 mM NaCl (Sigma-Aldrich), 10% glycerol (Thermo Fisher Scientific), 1 mM sodium orthovanadate (Sigma-Aldrich), 50 mM sodium fluoride (Sigma-Aldrich), 1 mM p-nitrophenyl phosphate (Sigma-Aldrich), and 1 mM PMSF (Sigma-Aldrich)]. The lysates were centrifuged and the concentration of protein in the cleared lysates was quantified using Bradford Protein Assay Reagent (Pierce, Waltham, MA, USA). Equal amounts of proteins were then separated on 10% or 12% SDS-PAGE gels and transferred onto nitrocellulose membranes (0.2 mm; GE Healthcare Life Science, Pittsburgh, PA, USA). Membranes were then blocked using 3-5% non-fat dry milk and 0.1% Tween-20 in Tris-buffered saline (TBS) and subsequently probed with primary antibodies in TBS containing 3% non-fat dry milk (Sigma-Aldrich) and 0.1% Tween-20 (Thermo Fisher Scientific). Antibody-antigen complexes were detected using goat anti-mouse IgG- or goat anti-rabbit IgG-peroxidase conjugates and enhanced chemiluminescence (ECL) detection kit (Amersham Bioscience, Piscataway NJ, USA).

Antibodies against ERK, MMP-9, MMP-2, c-Jun, E-cadherin, N-cadherin, Vimentin, and Actin were acquired from Santa Cruz Biotechnology (Dallas, TX, USA) and antibodies targeting p-JNK, p-p38, and p-ERK were taken from Cell Signaling (Danvers, MA, USA).

### Direct real-time analysis of cell migration

Real-time horizontal chemotaxis was detected using a KK chamber (Effector Cell Institute, Tokyo, Japan). This chamber is composed of an etched silicon substrate and a flat glass plate, together forming 2 compartments with a 5-µm-deep microchannel 38. Thermanox coverslips (Nalge-Nunc International, Rochester, NY, USA) were placed on the glass plates, the KK chamber was assembled in a stainless-steel holder, and 10% FBS was placed at one of the openings in the chamber. DMEM/F12 serum-free medium was placed in a contra-hole prior to 1 h incubation at 37°C. A charge-coupled device (CCD) camera was used to record the migration of serum toward the serum-free DMEM/F12 through the micro channel.

### In vitro Wound migration assay

Cells were grown to 90% confluence and then pretreated with mitomycin C (25 µg/ml; Sigma-Aldrich) for 30 min. A wound was created using a 2-mm pipette tip. Cells were rinsed briefly in PBS, fresh medium was added, and migration into the wound was monitored at the indicated time points by imaging at 40× magnification.

### Cell invasion assay

Cell invasion was assessed using the Cell Invasion Assay Kit (Chemicon, Temecula, CA, USA). Briefly, polycarbonate filters were coated with ECMatrix and placed in a transwell chamber. Medium, with or without 10% FBS, was added to the lower compartment of the chamber, then 3 × 10^5^ cells, suspended in DMEM medium without FBS, were added to the upper compartment. After 24 h incubation at 37°C, the filters were fixed with methanol and the cells that had invaded through the basement membrane were counted.

### Gelatin zymography

MCF10A, MCF10A-TMBIM6, MCF10A-TMBIM6-ΔC, MDA-MB231 and *TMBIM6* shRNA transfected MDA-MB231 cells were cultured in serum-free medium for 24 h. Conditioned medium was collected and centrifuged at 1,000 × g for 10 min to remove cell debris. The protein concentration was measured using BCA protein assay reagents (Pierce). Equal amounts of conditioned media were mixed with Laemmli non-reducing sample buffer, incubated for 15 min at room temperature, and separated by electrophoresis on 10% SDS-PAGE gels copolymerized with 1 mg/ml gelatin (Sigma-Aldrich). After electrophoresis, the gels were washed with 2.5% Triton X-100, twice, for 30 min; rinsed 3 times for 30 min in an incubation buffer containing 50 mM Tris-HCl buffer (pH 7.6), 5 mM CaCl2 (Sigma-Aldrich), 0.02% Brij-35 (Sigma-Aldrich), and 0.2% sodium azide (Sigma-Aldrich); and incubated overnight at 37°C in incubation buffer. Gels were stained with 0.5% Coomassie brilliant blue R-250 (Amresco LLC, Solon, OH, USA) solution containing 10% acetic acid (Sigma-Aldrich) and 20% methanol (Junsei, Tokyo, Japan) for 30 min and were then destained with 7.5% acetic acid solution containing 10% methanol. Areas of gelatinase activity were detected as clear bands against the blue-stained gelatin background.

### Analyses of breast cancer patient samples using database

To investigate the mRNA expression of TMBIM family in various cancer types and relative expression level in breast cancer compared to normal, ONCOMINE™ database (http://www.oncomine.org) was used. And relative TMBIM6 gene expression level in normal and breast cancers was analyzed from the ONCOMINE™ database. PrognoScan (http://dna00.bio.kyutech.ac.jp/PrognoScan/) and Kaplan-Meier plotter (http://kmplot.com/analysis) were used to investigate prognosis related to TMBIM6 expression level. Also TMBIM6 gene alteration in several types of cancer was analyzed using the cBioPortal (http://www.cbioportal.org). The significant threshold was adjusted to a Cox p-value < 0.05.

### Statistical analyses

All values are expressed as the means ± standard deviation (SD) and analyzed by using SPSS 16.0. Each value is the mean of at least 3 independent experiments for each group. Statistical significance of the differences between the 2 cell populations was determined using one-way analysis of variance (ANOVA) and the two-tailed Student's t-test. A p-value equal to or less than 0.05 was considered significant.

## Results

### Differential expression of the TMBIM family genes in breast cancer cells

**Figure 1:**
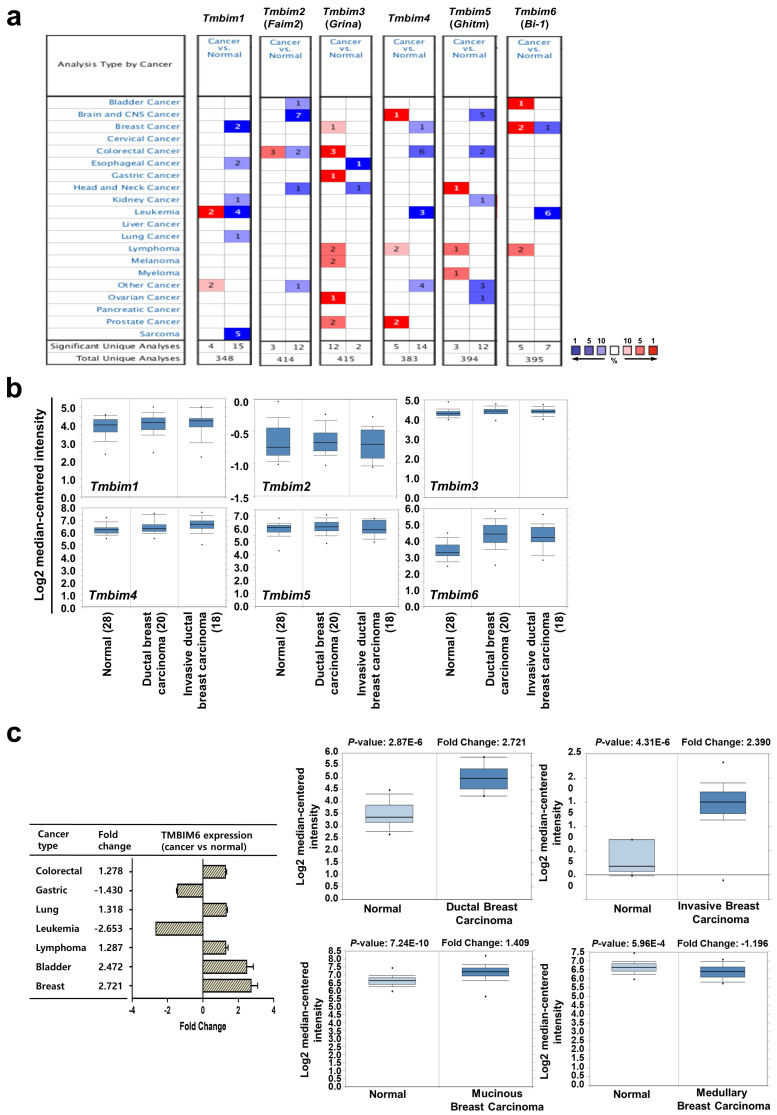

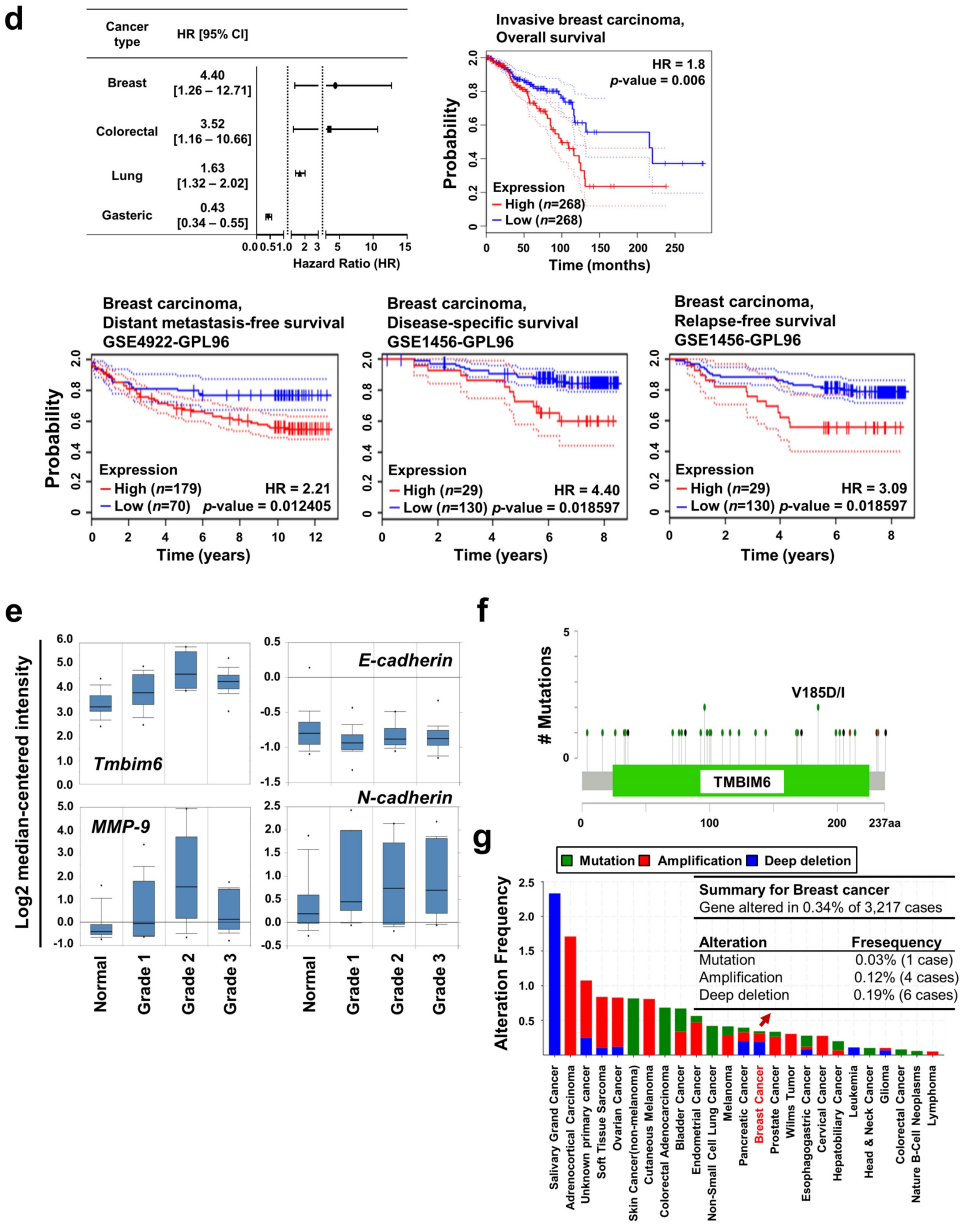
Relative expression analysis of TMBIM6 in breast cancer using the ONCOMINE™ database: (a) Global expression of the TMBIM family in various cancers versus normal tissues. This data showed a number of studies with more than two-fold changes in expression for tissue mRNA. The color code reflects the percentage range between the 10% groups of genes that have changed the most. Numbers indicate the amount of research reported by gene expression changes in the TMBIM family genes; (b) Relative levels of *TMBIM* family genes were analyzed in ductal and invasive ductal breast carcinomas using the Ma Breast 4 dataset in ONCOMINE ™ following these parameters: *p*-value = 1 × 10^-4^, Fold change = 2 and gene rank top 10%; (c) The fold change of TMBIM6 in various cancer types was identified by our analyses and expressed as a forest plot. Box plots comparing specific TMBIM6 expression in normal and in various breast carcinomas were derived from the ONCOMINE ™ database. The analysis was performed in ductal breast carcinoma from the Ma Breast 4 Statistics, invasive breast carcinoma from Finak Breast Statistics, mucinous breast carcinoma, and medullary breast carcinoma from Curtis Breast Statistics. (d) Hazard ratios in various cancer types were identified from our analyses and expressed as a forest plot. The survival curve comparing patient with high and low expression in breast carcinoma was plotted from the Kaplan Meier-plotter and PROGgene V2 database. Survival curve analysis was conducted using a threshold *Cox p*-value < 0.05; (e) Analysis of *TMBIM6, N-cadherin, E-cadherin*, and *MMP-9* expression according to breast cancer grade in Ma breast 4 dataset of the ONCOMINE ™ database. The image was downloaded from ONCOMINE ™; (f) Mutation diagram of TMBIM6 in different cancer types across protein domains from the cBioPortal; (g) The alteration frequency of TMBIM6 gene was determined using the cBioPortal. Cancer types including 100 samples and a change frequency of> 0.1% are displayed. The alteration frequency included mutation (green), amplification (red), or deep deletion (blue).

The most studied TMBIM family to date is TMBIM6, which has been reported to be closely associated with cancer progression 7, 39, 40. The TMBIM family of proteins includes *TMBIM1* (Responsive to centrifugal force and shear stress gene 1 protein, Recs1), *TMBIM2* (life guard, *Lfg*), *TMBIM3* (Glutamate receptor ionotropic NMDA protein 1, *Grina*), *TMBIM4* (Golgi antiapoptotic-associated protein, *Gaap*), *TMBIM5* (Growth hormone-inducible transmembrane protein, *Ghitm*), and *TMBIM6* 39. We systemically analyzed the mRNA expression profiles of the TMBIM family between normal tissues and various tumor tissues using ONCOMINE™ cancer microarray database (www.oncomine.org) (Fig. 1a). Interestingly, *TMBIM1, TMBIM2, TMBIM4*, and *TMBIM5* were found to decrease in many types of cancer, but *TMBIM3* and *TMBIM6* were significantly increased in this analysis. Especially, in breast cancer, expression of *TMBIM6* was up-regulated compared to in normal tissue (Fig. 1a). However, all other TMBIM family genes were expressed higher in the other cancer types.

Using the ONCOMINE™ database, we compared the expression patterns of the *TMBIM* family genes (*TMBIM1-6*) in normal and cancer tissues. Notably, ductal or invasive ductal breast carcinoma showed significantly higher *TMBIM6* expression than normal breast tissue in the Ma breast 4 datasets (Fig. 1b) 41. Moreover, we found that *TMBIM6* was up-regulated in bladder, lung, lymphoma, colorectal, and breast cancers, but specially increased in ductal or invasive breast carcinoma compared to in their normal tissue (Fig. 1c, Figure S1) 41-50. In Fig. 1d (left upper panel), we summarized the prognostic value of TMBIM6 expression in various cancers from PrognoScan database with Cox proportional hazards regression analysis. Furthermore, the Kaplan Meier-plot and PrognoScan revealed that high expression of TMBIM6 was not beneficial for the prognosis of breast cancer (Fig. 1d, Figure S2).

We found that expression of *TMBIM6* was upregulated with histological grade compared to that in normal tissues (Fig. 1e). Furthermore, the expression of *MMP-9*, and *N-cadherin* increased with histological grade, but the expression of *E-cadherin* decreased. This result indicated a correlation between *TMBIM6* expression and invasion in breast cancer.

To analyze TMBIM6 mutations and copy number alternations (CNAs) in various cancer types, we used the cBioPortal database to investigate mutations in various parts of TMBIM6, particularly in V185D / I (Fig. 1f). The results showed that TMBIM6 mutations were increased in several cancer types, particularly in breast and salivary gland cancer with alteration frequencies. Data were observed in various cancer type studies, with frequency of changes of more than 0.1% in over 100 samples for each cancer type in the dataset (Fig. 1g). The rate of alteration was 2.33 - 0.05% in decreasing order in the various cancer types and the highest alteration frequency was observed in salivary gland cancers. However, we have focused on breast cancer because of various alterations. We observed the alteration frequency of at least 0.34% in breast cancer with 3,217 samples (Fig. 1g).

### Knockdown of TMBIM6 decreases cell proliferation, migration, and EMT

To investigate the role of *TMBIM6* in breast cancer cells, we analyzed *TMBIM6* and EMT markers expression in a panel of MCF10A cells as human immortalized normal breast epithelial cells and MCF-7, MDA-MB231, and MDA-MB468 as breast cancer cell lines with varying prognosis. MCF-7 cells are characterized as estrogen receptor (ER)-positive/ progesterone (PgR)-positive/ human epidermal growth factor receptor (HER2)-negative, luminal subtype, while MDA-MB468 and MDA-MB231 cells are known as triple-negative breast cancer (TNBC), ER-negative/ PgR-negative/ HER2-overexpression, basal subtype. According to several studies, triple-negative show more aggressiveness than luminal subtypes 51, 52.

**Figure 2:**
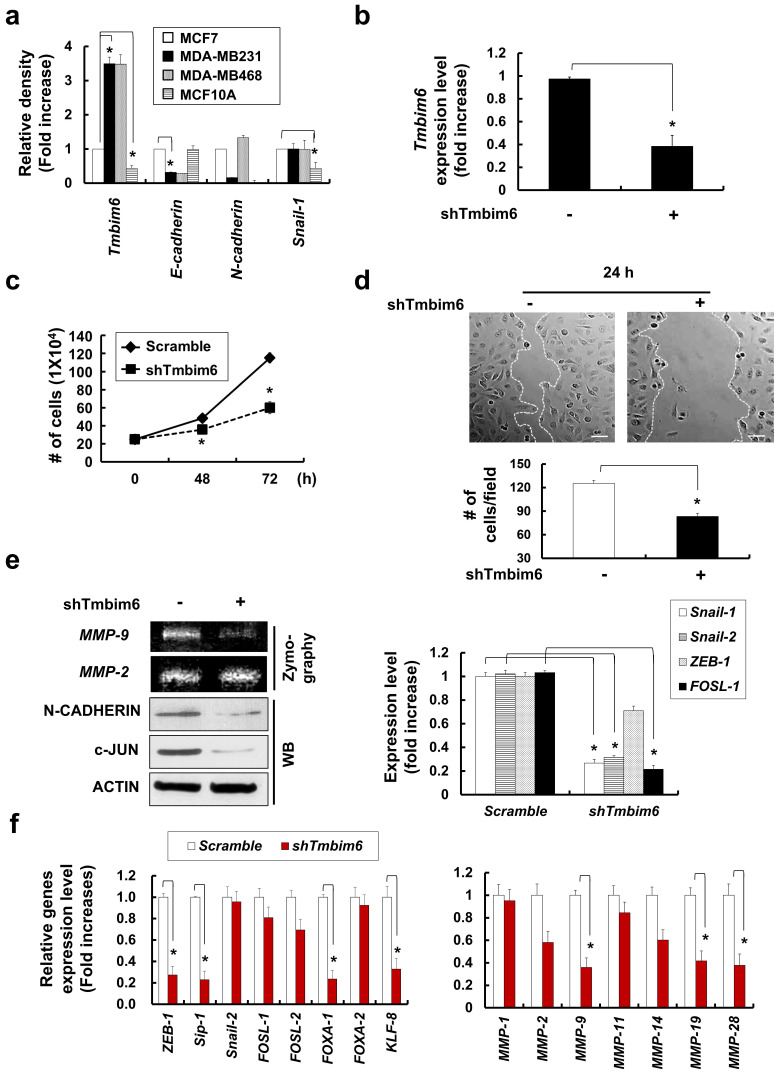
Effects of cell growth, and EMT-related gene expression in *shTMBIM6* MDA-MB231 cells: (a) *TMBIM6*, *E-cadherin*, *N-cadherin*, and *Snail-1* expression detected by RT-PCR in indicated cells. Densitometry quantitation of PCR bands was performed after normalization to *GAPDH* as an internal control. *p < 0.05; (b) MDA-MB231 cells were transfected with scramble or TMBIM6-specific shRNA. Endogenous *TMBIM6* mRNA was determined by qRT-PCR; (c) Cell counts (mean ± SD, n = 3) of scramble or *shTMBIM6* MDA-MB231 cells seeded at 2 × 10^5^ cells; (d) Migration of MDA-MB231 and *shTMBIM6* MDA-MB231 cells into wounds made in the monolayer. Photographs of the cultures were taken immediately after wounding (0 h) and after 24 h in culture. Lower panel graph: number of migrating cells between two white lines (mean ± SD, n = 3). Scale bar: 100 μm; (e) Left panel, MDA-MB231 and *shTMBIM6* MDA-MB231 cells were used to determine activity of MMP-9 and MMP-2 by gelatin zymography. Right panel, total RNA was used to examine the expression of *Snail-1*, *Snail-2*, *ZEB-1*, and *FOSL-1* in cells by qRT-PCR; (f) Based on RNA seq. analysis, a transcriptional profile of the EMT-related transcription factors (*ZEB-1*, *Sip-1*, *Snail-1*, *FOSL-1*, *FOSL-2*, *FOXA-1*, *FOXA-2*, and *KLF-8*) (left) and migration-related MMPs (*MMP-1*, *MMP-2*, *MMP-9*, *MMP-11*, *MMP-14*, *MMP-19*, and *MMP-28*) (right) genes were presented. Compared with TMBIM6 expression in the control (scramble) shRNA-transduced cells, specific downregulation of TMBIM6 expression was achieved in the *shTMBIM6* MDA-MB231 cells. Error bars represent the mean ± SD of each gene. *p<0.06

Similarly, we confirmed that expression level of the *TMBIM6* and EMT markers, *N-cadherin* and *Snail-1* in MDA-MB231 and MDA-MB468 was higher than MCF7 and MCF10A cell lines. In present study, endogenous *TMBIM6* expression was significantly upregulated in highly tumorigenic cell lines, whereas expression was negative or low in non-tumorigenic mammary epithelial cell lines (Fig. 2a). Based on these results, we suppressed TMBIM6 using short hairpin RNA (shRNA) and compared the expression with cells transformed by scrambled shRNA. We confirmed specific downregulation of *TMBIM6* expression in *shTMBIM6* MDA-MB231 cells (Fig. 2b). Knockdown of *TMBIM6* expression decreased cell proliferation (Fig. 2c) and migration (Fig. 2d). MMP-9 activity, expression of EMT markers, and EMT-related transcription factors were also significantly decreased upon *shTMBIM6* MDA-MB231 cells (Fig. 2e).

Using RNA-seq. analysis, we evaluated the expression of EMT-related transcription factors and migration-related MMPs and determined that they were decreased in *shTMBIM6* MDA-MB231 cells (Fig. 2f).

### *TMBIM6* knockdown suppresses MAPK/ERK signaling by inhibiting the expression of *miR-181a*

**Figure 3:**
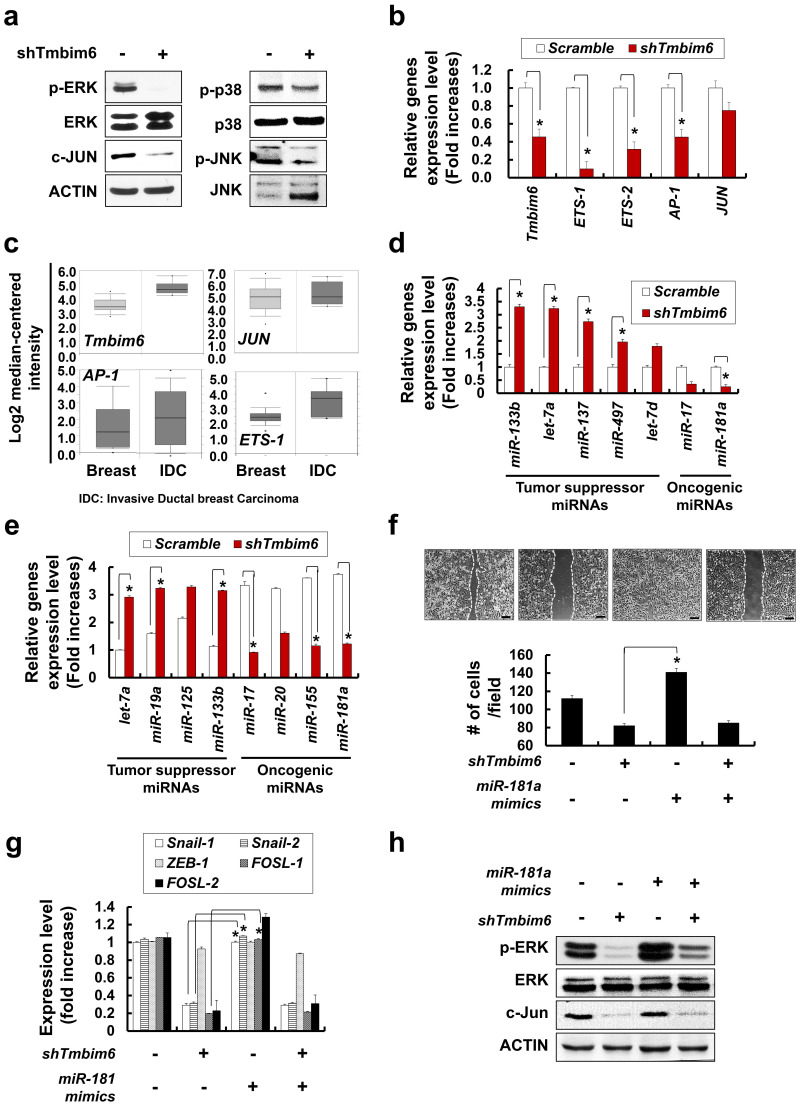
Effects of *TMBIM6* knockdown on ERK activation and *miR-181a* expression in MDA-MB231 cells. (a) Cell extracts were subjected to western blotting to detect phosphor-ERK and c-Jun or actin and total ERK as loading controls. (b) Based on RNA seq. analysis, a transcriptional profile of *TMBIM6*, *ETS-1*, *ETS-2*, *AP-1* and *JUN* (*c-Jun*) genes is presented. (c) Comparison of *TMBIM6*, *AP-1*, *ETS-1* and *JUN* mRNA expression levels in normal breast tissue with those in invasive ductal breast carcinoma based on the Ma breast 4 dataset of the ONCOMINE ™ database. (d) Based on RNA seq. analysis, a transcriptional profile of tumor suppressor miRNAs (*miR-133b*, *let-7a*, *miR-137*, *miR-497*, and *let-7d*) and oncogenic miRNAs (*miR-17* and *miR-181a*) genes is presented. (e) The mRNA expression level of *let-7a*, *miR-19a*, *miR-125*, *miR-133b*, *miR-17*, *miR-20*, *miR-155*, and *miR-181a* were analyzed in *shTMBIM6* MDA-MB231 cells. (f) Migration in the indicated cells was analyzed using a migration assay. The number of cells in the enclosure was enumerated at 24 h. Scale bar: 200 μm; (g) The mRNA expression level of *Snail-1*, *Snail-2*, *ZEB-1*, *FOSL-1*, and *FOSL-2* were analyzed in *shTMBIM6* MDA-MB231 cells. (h) Cell extracts were subjected to western blotting to detect phosphor-ERK and c-Jun or actin and total ERK as loading controls.

We next investigated the underlying intracellular signaling mechanism of the *TMBIM6* knockdown phenotypes in breast cancer cells. We observed that MAPK/ERK signaling pathway was inactivated in response to *TMBIM6* knockdown, while the activity of the JNK and p38 was not altered (Fig. 3a). Using RNA-seq. analysis, *TMBIM6* knockdown led to decreased expression of MAPK-related transcription factors, such as E-twenty-six (*ETSs*), activator protein (*AP-1*), and *JUN* (Fig. 3b). Furthermore, ONCOMINE™ database analyses supported the observation that *TMBIM6* expression correlates with MAPK-related transcription factors (Fig. 3c).

Several studies have indicated that miRNAs can promote the migration and metastasis of cancer cells 53-55, thus, we also investigated miRNAs expression in *TMBIM6* knockdown cells. Using RNA-seq. analysis, *TMBIM6* knockdown led to decreased expression of oncogenic miRNAs, such as *miR-17* and *miR-181a* (Fig. 3d). However, the expression of tumor suppressor miRNAs (*miR-133b*, *let-7a*, *miR-137*, *miR-497*, and *let-7d*) was significantly increased in *TMBIM6* knockdown cells. Furthermore, we observed decreases in the expression level of oncogenic miRNAs, such as *miR-17*, *miR-20*, *miR-155*, and *miR-181a* in *TMBIM6* knockdown cells (Fig. 3e). Especially, we found that the expression of *miR-181a* and *miR-17* were significantly reduced in *TMBIM6* knockdown cells. Previous reports have shown abnormally elevated expression of miR-181a in cancers, suggesting that *miR-181a* performs a tumor-promoting role in these environments 32- 34. In this context, we investigated the effects of activating the MAPK/ERK signaling pathway by treating *miR-181a* mimics in *TMBIM6* knockdown cells. The *miR-181a* mimics could not change the *shTMBIM6*-mediated decrease in cell migration (Fig. 3f), expression of EMT-related transcription factors (Fig. 3g), ERK phosphorylation and c-Jun level (Fig. 3h). These results indicated that regulation of cell migration and MAPK/ERK signaling is related to TMBIM6-mediated *miR-181a* expression.

### Overexpression of *TMBIM6* leads to increased EMT gene expression, migration and invasion in non-tumorigenic mammary epithelial MCF10A cells

**Figure 4:**
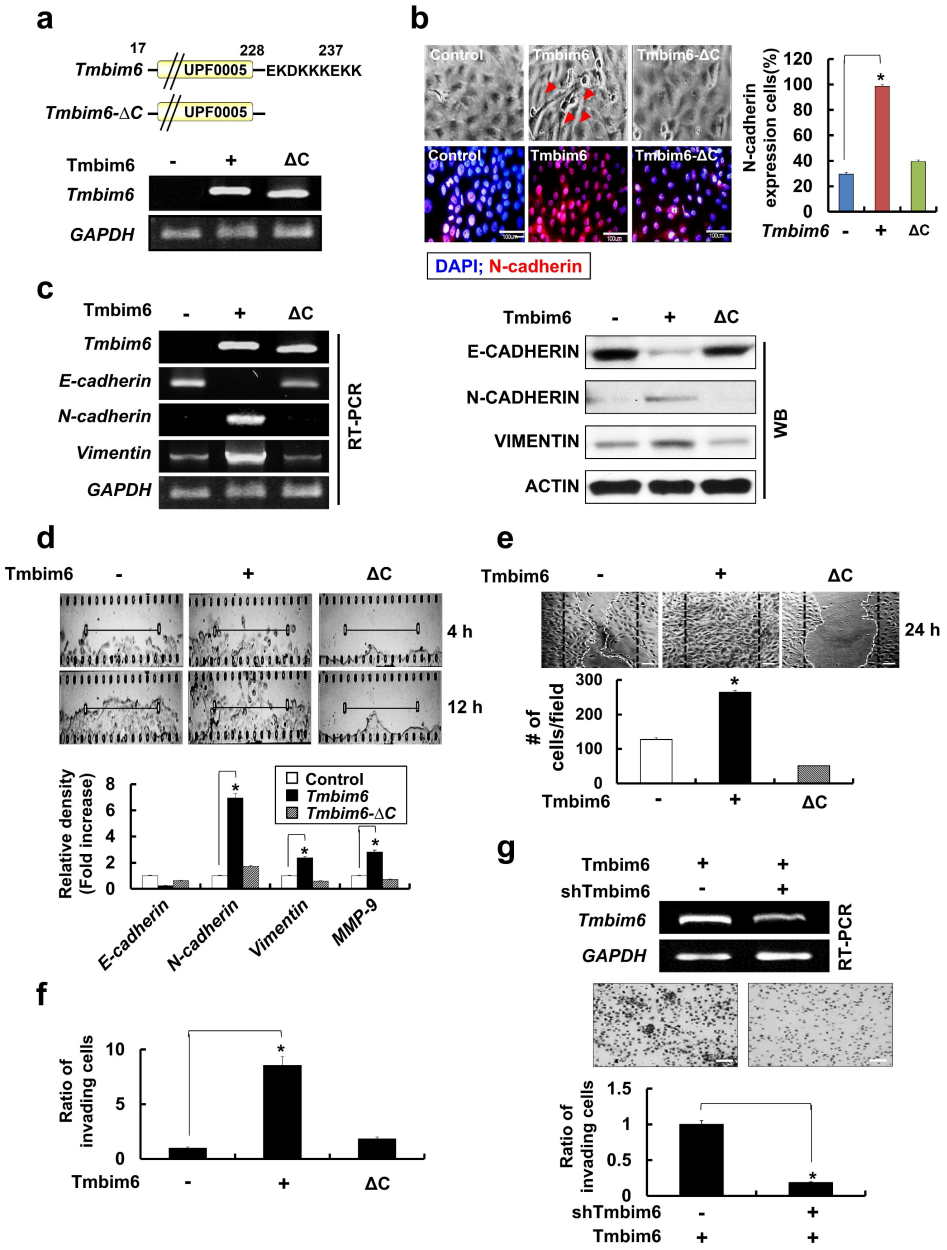
Morphological Changes and Downregulation of E-cadherin by *TMBIM6* Overexpression in MCF10A Cells. (a) Upper panel: structure of TMBIM6 and TMBIM6-∆C plasmids. Lower panel: exogenous expression of *TMBIM6* and *TMBIM6*-∆C, as detected by RT-PCR. (b) Left panel: cell morphology of each cell line was observed by phase contrast microscopy. Red arrows represent overgrowth of MCF10A cells. Right panel: immunofluorescence staining of N-cadherin in each cell line and count of N-cadherin stained cells. The red signal represents N-cadherin staining, and the blue signal represents nuclear DNA staining by DAPI. Scale bar: 100 µm; (c) Analysis of the expression of *TMBIM6*, *E-cadherin*, *N-cadherin*, and *Vimentin* by RT-PCR and western blotting in the indicated cells. Actin and *GAPDH* were used as loading controls. (d) Upper panel, Cells were subjected to *in vitro* direct real-time analysis of cell migration using a KK chamber. After aligning the cells to the edge of the microchannel (at the bottom of each figure), by pulling out the medium from the opposite compartment (located above each figure, not seen), the medium was replaced, to the top level of the common space. Migration of cells in the microchannel was recorded at 10 min intervals onto a computer hard disk using a CCD camera. The rectangular structure in the center of the channel is one of 2 position markers in the channel. Bar, 500 µm. Lower panels: Expression levels of *E-cadherin*, *N-cadherin*, *Vimentin* and *MMP-9* during migration in each cell. (e) The migration of MCF10A, MCF10A-*TMBIM6,* and MCF10A-*TMBIM6*-∆C cells into wounds made in the monolayer. Photographs of the cultures were taken immediately after 24 h in culture. Scale bar: 200 μm; (f) Cells were incubated in invasion chambers for 24 h, and the number of invaded cells was counted. Invasive cells that migrated through the ECM layer and adhered to the bottom of the basement membrane were counted in 13 arbitrary visual fields per sample at 200× magnification, and averaged. Graphs depict means ± S.E. of triplicate samples. (g) Knockdown of *TMBIM6* by specific shRNA was confirmed by RT-PCR. Control cells were transfected with a control pSilencer vector. The invasion of shTMBIM6-transfected cells was examined using an in vitro invasion assay. Scale bar: 100 μm. Statistically significant differences compared to the control are indicated by *, where p < 0.01 in a Student's t-test.

To confirm the function of TMBIM6 in regulating the MAPK/ERK signaling pathway, we overexpressed *TMBIM6* and non-functional mutant (*TMBIM6-ΔC*) in the MCF10A cells. TMBIM6-ΔC was prepared through the deletion of the last nine C-terminal amino acids of the TMBIM6 protein. We were able to detect the apparent expression of exogenous *TMBIM6* and *TMBIM6-ΔC* transcripts in TMBIM6 or TMBIM6-ΔC MCF10A cells, respectively (Fig. 4a). Overexpression of *TMBIM6* led to cell morphological changes, and specifically cells became elongated and spindle-like shaped (Fig. 4b). MCF10A cells had cuboidal-shaped morphology, similar mesenchymal-like cells. Since the loss of epithelial morphology and the acquisition of mesenchymal characteristics are typical for carcinoma cells during tumor progression 56, 57, we also examined the expression of common EMT markers in *TMBIM6*-overexpressing cells. As shown in Fig. 4b and 4c, N-cadherin expression was significantly increased in *TMBIM6*-overexpressing cells, indicating that TMBIM6 may be involved in the loss of epithelial cell character. Moreover, E-cadherin expression was dramatically decreased upon the *TMBIM6* overexpression (Fig. 4b and 4c), suggesting that TMBIM6-induced morphological changes may be involved in EMT.

The development of EMT features has been implicated in the invasion, metastasis, and, ultimately, transformation of cancer cells 58. To investigate the effects of *TMBIM6* overexpression on the invasive and migratory capacities, we performed invasion and migration assays. To evaluate the effects of *TMBIM6* overexpression on migration, KK chamber analysis was used to detect real-time horizontal migration. *TMBIM6*-overexpression resulted in enhanced directional and active migration compared to those in both control and *TMBIM6-ΔC*-expressing cells (Fig. 4d, upper panel). Concurrently, EMT-related genes, *N-cadherin*, *Vimentin*, and *MMP-9* were upregulated in *TMBIM6*-overexpressing cells (Fig. 4d, lower panel). Wound healing assay was also performed to determine the role of TMBIM6 in modulating cell motility. Overexpression of *TMBIM6* was able to migrate more efficiently into the wound region, compared to those expressing *TMBIM6-ΔC* (Fig. 4e). Furthermore, we found that overexpression of *TMBIM6* led to significantly induced cells invasion (Fig. 4f).

Next, we attempted to confirm the role of TMBIM6 in cell invasion and migration using shTMBIM6. This approach was shown to effectively knockdown the expression of *TMBIM6* in *TMBIM6*-overexpressing cells, as shown by RT-PCR analysis (Fig. 4g, upper panel). The silencing of *TMBIM6* expression relieved the invasive capacity of *TMBIM6*-overexpressing cells (Fig. 4g, lower panel).

### *TMBIM6* overexpression markedly upregulates MMP-9 expression and activation

**Figure 5:**
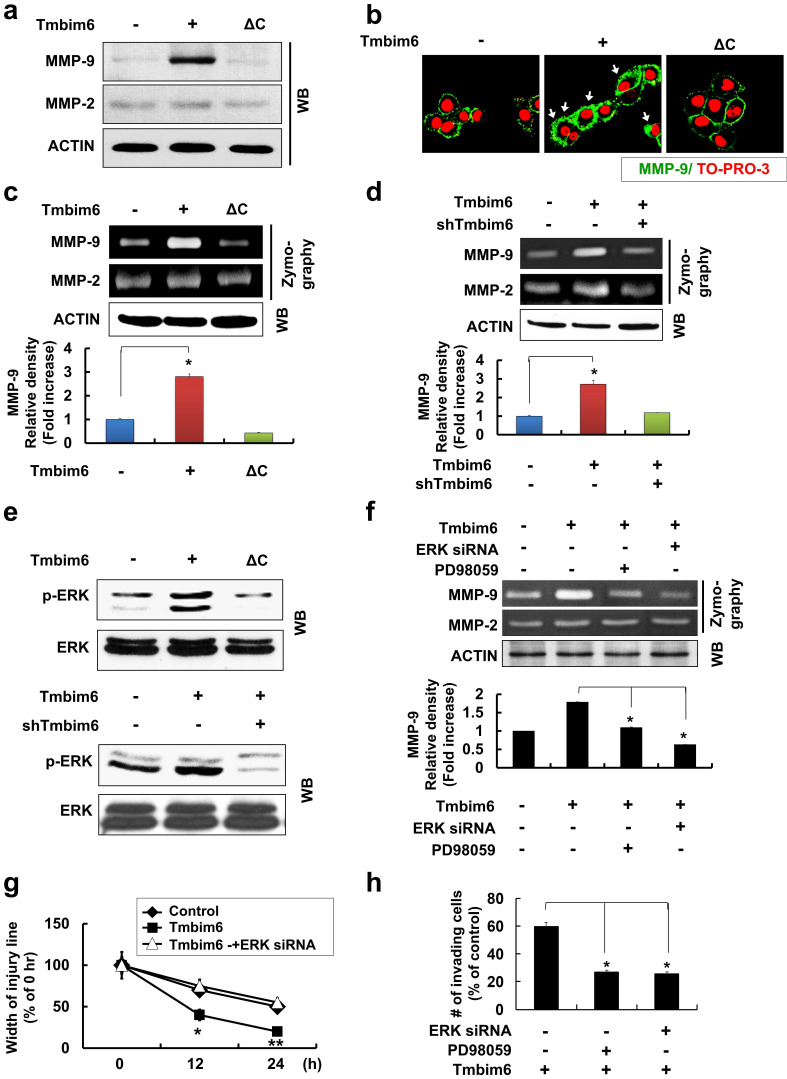
Critical roles of the MAPK / ERK signaling pathway for TMBIM6-induced MMP-9 upregulation, migration and invasion in MCF10A cells. The expression of MMP-9 in conditioned medium was examined by (a) western blot analysis and (b) immunocytochemical analysis (green: MMP-9 and red: TO-PRO-3 for counterstaining the nuclei). Arrows indicate the upregulation of MMP-9 expression under TMBIM6 overexpression; (c, d) The gelatinolytic activity of secreted MMP-9 from conditioned media was determined by gelatin zymography in indicated cells. Densitometry quantitation of MMP-9 bands was performed after normalization against ACTIN as an internal control. (e) The levels of activated ERK in the indicated cells were determined by western blot analysis of whole-cell lysates using anti-phosphor-ERK and anti-ERK antibodies. (f) Upper panel, MCF10A-TMBIM6 cells were transfected with ERK siRNA, or treated with a MEK inhibitor (PD98059) and conditioned media were collected 24 h later. Lower panel: densitometry quantitation of MMP-9 bands was carried out after normalization against actin as an internal control. (g) Cell motility was examined under light microscope (40× magnification) at the indicated time points. Migratory ability is presented as the percentage of migrating cells with respect to the total number of cells. (h) MCF10A-TMBIM6 cells were treated with PD98059 or transfected with ERK siRNA, and cells were subjected to an in vitro invasion assay 24 h later. The results presented are means ± S.E. of triplicates experiments. *, p < 0.01; **, p < 0.05 versus the control.

The degradation of type IV collagen, the major structural collagen of the extracellular matrix, by MMP-2 and/or MMP-9, is often associated with tumor invasion and metastasis 59. *TMBIM6* overexpression resulted in a marked induction in MMP-9, but not MMP-2 expression (Fig. 5a), and the observation was confirmed by immunocytochemistry (Fig. 5b). Additionally, MMP-9 activity, assessed by a gelatin zymography assay, was increased in the conditioned media of *TMBIM6*-overexpressing cells compared to that of control cells, while MMP-2 activity was unchanged (Fig. 5c). Conversely, TMBIM6-induced MMP-9 activity could be significantly downregulated by *TMBIM6* knockdown (Fig. 5d). These results demonstrate that TMBIM6 might induce invasive and migratory phenotypes through the upregulation of MMP-9 expression and activity.

### MAPK/ERK signaling is critical for TMBIM6-induced MMP-9 upregulation, and cell invasion and migration

Next, we investigated the signaling pathways involved in the upregulation of MMP-9 activity, cell invasion, and migration in response to *TMBIM6* overexpression. We found that the MAPK/ERK signaling pathway was activated in response to *TMBIM6* overexpression (Fig. 5e, upper panel), while the activities of JNK and p38 were not altered (data not shown). Moreover, *TMBIM6* knockdown resulted in a significant reduction in ERK phosphorylation levels (Fig. 5e, lower panel). To address the functional role of MAPK/ERK in the regulation of MMP-9 expression and the development of invasive and migratory phenotypes in *TMBIM6*-overexpressing cells, we used both a specific inhibitor of the MAPK/ERK pathway (PD98059) and ERK-specific siRNA. Inhibition of MAPK/ERK by PD98059 or ERK-siRNA resulted in significant suppression of MMP-9 activity, as measured by gelatin zymography (Fig. 5f). Moreover, treatment with PD98059 or transfection with ERK-siRNA was also significantly inhibited both invasion and migration in *TMBIM6*-overexpressing cells (Fig. 5g and 5h). Collectively, our data demonstrate that the observed TMBIM6-induced upregulation of MMP-9 expression and increases in invasion and migration in non-tumorigenic epithelial MCF10A cells are dependent on the activation of the MAPK/ERK signaling pathway.

### *TMBIM6*-mediated MAPK/ERK activation leads to increased *FOSL-1*- and *c-Jun*-dependent *Snail-1* and *Snail-2* expressions

Several studies have identified a variety of essential transcription factors, such as specificity protein 1 (Sp1) and protein kinase C1 (PKC1), for the expression of *TMBIM6* 60. Some studies have demonstrated that several transcription factors (*Snail-1*, *Snail-2*, *ZEB-1*, *SIP1*, and *Twist*) act as transcriptional repressors of mesenchymal-epithelial transition (MET) marker, E-cadherin 58. These proteins also mediate the upregulation of genes and proteins related to cell invasion and motility such as Vimentin, Fibronectin, and MMP family proteins.

**Figure 6:**
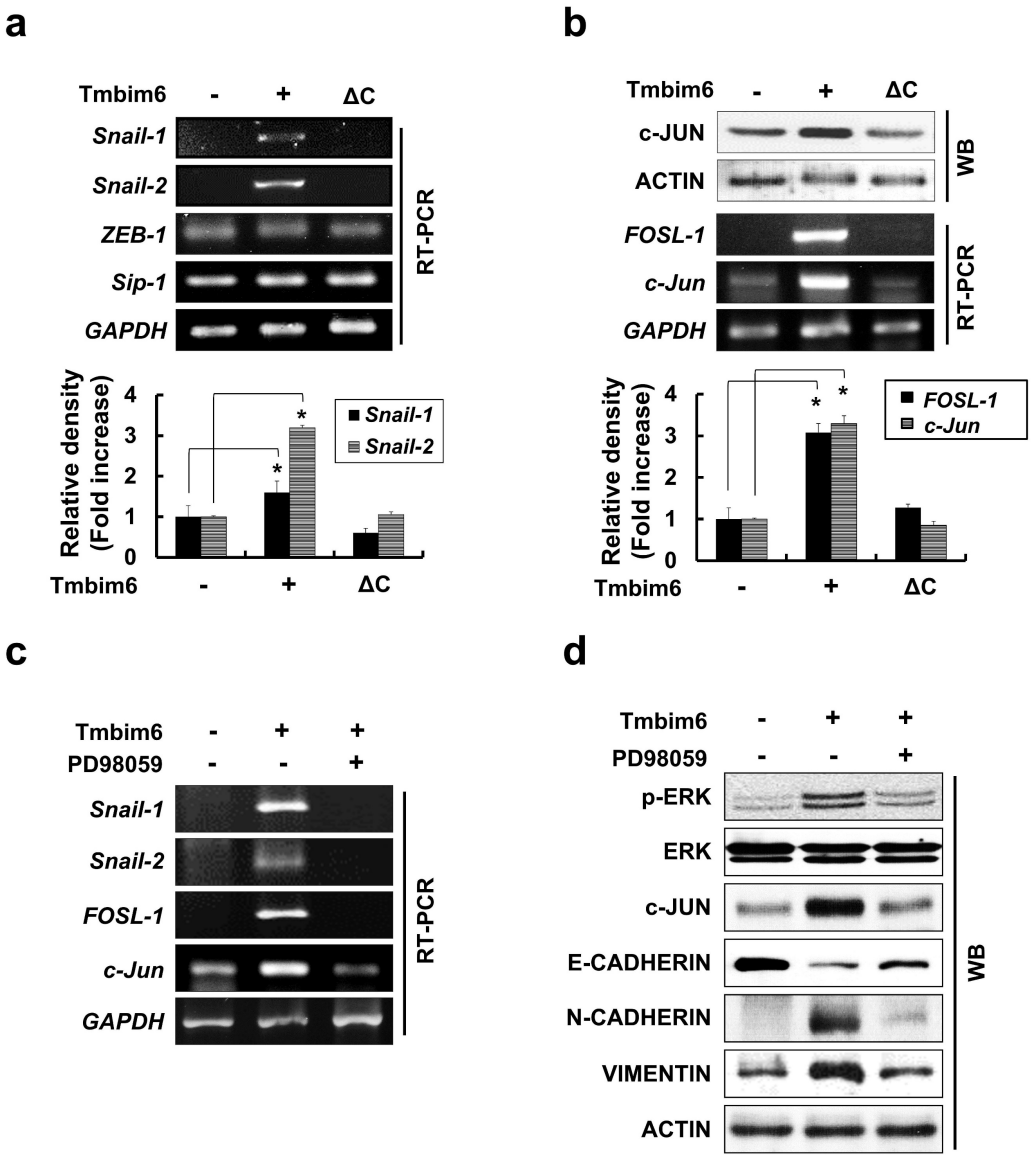
Enhancement of Snail-1 and Snail-2 expression of FOSL-1 and c-Jun by TMBIM6-mediated ERK activation. (a) The expression of *Snail-1*, *Snail-2*, *ZEB-1*, *Sip-1*, and *GAPDH* genes were analyzed in the indicated cells by RT-PCR. Densitometry quantitation of *Snail-1* and *Snail-2* PCR bands was carried out after normalizing against *GAPDH* as the internal control. *p < 0.05 (b) Western blot analysis was conducted using specific antibodies targeting c-JUN and ACTIN, and the mRNA levels of *FOSL-1* and *c-Jun* transcripts in each cell line were assessed by RT-PCR. (c) TMBIM6-expressing MCF10A cells were incubated with PD98059 for 24 h. (d) Cell lysates were then analyzed for the expression of phosphor-ERK, ERK, c-JUN, E-CADHERIN, N-CADHERIN, VIMENTIN, and ACTIN by western blot analysis. *p < 0.05.

In our study, E-cadherin was clearly down-regulated in *TMBIM6*-overexpressing cells compared to control cells (Fig. 4c). Therefore, we examined whether *TMBIM6* overexpression affects the expression of *Snail-1*, *Snail-2*, *Sip1*, and *ZEB-1*. As shown in Fig. 6a, *Snail-1* and *Snail-2* mRNAs were significantly upregulated in *TMBIM6*-overexpressing cells compared to control cells. However, very little changes were observed in *ZEB-1* and *Sip1* mRNA levels in both the control and *TMBIM6*-overexpressing cells, indicating that overexpression of *TMBIM6*-induced *Snail-1* and *Snail-2* act as the suppressors of *E-cadherin* transcription. We further analyzed the molecular mechanisms through which *TMBIM6* induces *Snail-1* and *Snail-2* expression and investigated the potential involvement of candidate transcription factors. Since *AP-1* transcription factor complexes have been shown to play a critical role in the expression of Snail family proteins via multiple mechanisms, we examined the expression of the main proteins that form AP-1 complexes in breast cancer cells, including c-Jun, c-Fos, and FOSL-1. Our data demonstrated that both mRNA and protein expression of FOSL-1 and c-Jun were markedly increased in *TMBIM6*-overexpressing cells compared with control cells (Fig. 6b). Notably, the treatment with PD98059 completely abolished *FOSL-1* and *c-Jun* expression (Fig. 6c and 6d), indicating that MAPK/ERK activation in *TMBIM6*-overexpressing cells is important for c-Jun/FOSL-1-mediated *Snail-1* and *Snail-2* expression.

### Inhibition of* miR-181a* decrease migration, expression of EMT-related transcription factors and p-ERK levels

**Figure 7:**
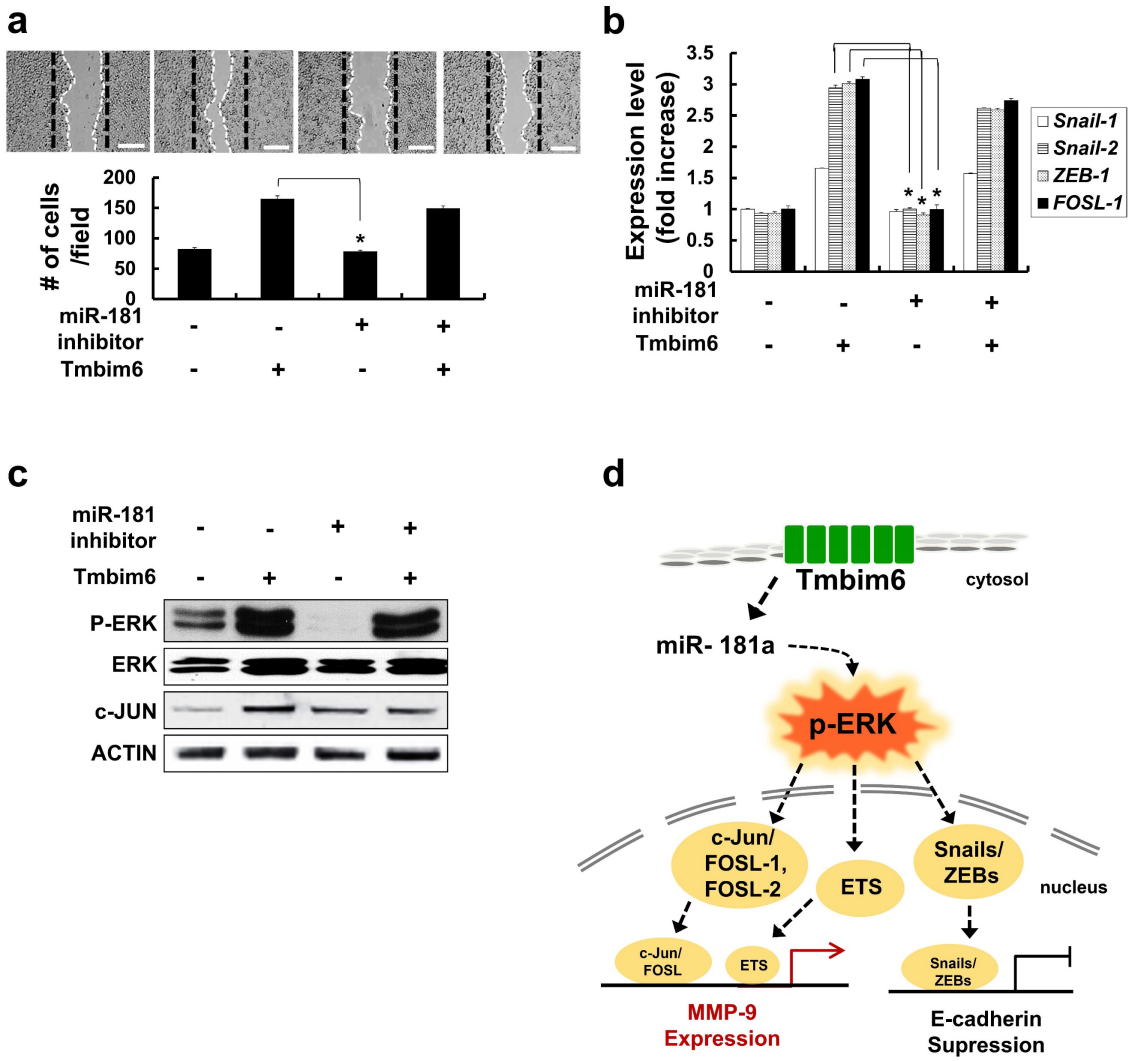
Inhibition of Snail-1 and Snail-2, ZEB-1 and FOSL-1 expression in miR-181a inhibitor-treated TMBIM6-overexpressing MCF10A cells. (a) Migratory capacity of the indicated cells was analyzed using a migration assay. The number of cells in the enclosure was enumerated at 24 h. Scale bar: 500 μm; (b) the mRNA expression levels of *Snail-1*, *Snail-2*, *ZEB-1*, and *FOSL-1* were analyzed in the indicated cells; (c) Cell extracts were subjected to western blotting to detect p-ERK and c-JUN or ACTIN and total ERK as loading controls; (d) Schematic illustration of TMBIM6-mediated miR-181a was enhanced to breast cancer cell migration and invasion.

To investigate the effects of *miR-181a* expression on the migrative capacity of TMBIM6 overexpression, a migration assay was performed using *miR-181a* inhibitors. We confirmed that there was little change in migration and expression levels of *Snail-1*, *Snail-2*, *ZEB-1*. *FOSL-1* by transfection of *miR-181a* inhibitors into normal mammary epithelial cells. However, inhibition of miR-181a suppresses migration and down-regulates EMT-related transcription factors, in *TMBIM6*-overexpressing cells (Fig. 7a and 7b). Moreover, a decrease in p-ERK and c-JUN levels was shown by *miR-181a* inhibitor (Fig. 7c).

## Discussion

Breast cancer is one of the most commonly diagnosed types of cancer in women. Since metastasis is the main cause of death from breast cancer, the development of therapeutic approaches that regulate breast cancer metastasis has been an important area of research. The novel anti-apoptotic membrane protein, TMBIM6 has been shown to be overexpressed in various cancers, including lung 7, 61, prostate 8, and breast 9, 62. Significantly, specific down-regulation of TMBIM6 by RNA interference in prostate cancer cells has been reported to result in cell death 8. Various reports have suggested that miR-181a play important roles in tumor invasion and metastasis 26, 59. In this study, we demonstrated that upregulation of TMBIM6-induced miR-181a may play an important role in the migration and invasion of breast cells through the MAPK / ERK signaling pathway.

The EMT, a critical process required for embryogenesis, was shown to be related to cell migration and invasion 56, 57, 63. We show that TMBIM6 was highly expressed in breast cancer samples using mRNA expression assays and the ONCOMINE™ database. Moreover, we observed a significant reduction in N-cadherin expression upon knockdown of *TMBIM6* of MDA-MB 231 cells, suggesting that the EMT plays a role in TMBIM6-inhibted invasion and migration in these cells. Furthermore, we investigated that the expression of miR-181 family genes was decreased after knockdown of *TMBIM6* in MDA-MB 231 cells, leading to inactivation of the MAPK/ERK pathway, while the JNK and p38 pathways were unaffected. Also, we observed a marked induction in the N-cadherin expression and a significant reduction in the E-cadherin expression by *TMBIM6* overexpression. Significant increases in MMP-2 and MMP-9 expression have also been reported in many types of human cancers, as well as in vitro and in vivo models of cancer, providing evidence for a strong correlation between the expression of proteins and cancer invasion or metastasis 16, 64. Given that the role of MMPs in the progression and metastasis of mammary tumors has been well demonstrated. It is possible that the TMBIM6-induced progression and metastasis of breast cancer can be mediated through MMP-2 and/or MMP-9.

In this study, we provided in vitro evidence that the TMBIM6 overexpression greatly increased the secretion of active MMP-9 in MCF10A cells. In contrast, increased activation of MMP-2 was not observed in these cells, suggesting that TMBIM6 specifically affects the expression of MMP-9. Further investigation of the potential mechanisms through which TMBIM6 induces MMP-9 upregulation in MCF10A cells is necessary to determine if this observation is a direct result of gene transcription.

To increase the success rate of various cancer therapies requires a clear understanding of the mechanisms that promote metastasis. Several factors have been shown to be involved in the regulation of cancer metastasis, including those involved in MAPK signaling pathway 19, 23, 65. Previous reports have shown that the ERK signaling pathway is an attractive target for therapeutic intervention due to its integral role in the regulation of proliferation, invasiveness, and tumor survival 19, 23, 66. Several studies using siRNAs and pharmacologic inhibitors have demonstrated the importance of the MAPK/ERK signaling in cancer cells, and several agents that target this pathway are undergoing clinical testing, are promising 67. The current study demonstrated that stable overexpression of TMBIM6 enhances activation of the MAPK/ERK pathway in MCF10A cells but does not affect the activation of p38 and JNK signaling pathways, suggesting that the MAPK/ERK signaling pathway, not the p38 and JNK signaling pathways, is involved in TMBIM6-induced invasive/migratory phenotypes. Similar results were reported using LY6K-overexpressing cells, where cell invasion was found to be mediated by the ERK pathway 68. For the first time, we have shown that the ERK pathway is required for TMBIM6-induced invasion and migration in MCF10A cells. Moreover, ERK activation in response to TMBIM6 overexpression led to increases in MMP-9 activity and the expression of the E-cadherin-suppressor proteins, Snail-1 and Snail-2 (Fig. 7d). Our data clearly indicate that the ERK-induced Snail-1/Snail-2 expression signaling pathway is crucial for TMBIM6-induced MCF10A cell invasion. However, further investigation is still required to elucidate the role of ERK activation in the TMBIM6-mediated mammary tumor progression.

Given that TMBIM6 is one of the most important oncogenes in human breast cancer and an attractive therapeutic target, our findings may provide a molecular basis for the role of TMBIM6 in the promotion of breast cancer progression. The current study is also a remarkable report describing another new molecular mechanism related to the TMBIM6-induced migration and invasion and has identified the key molecules and signaling pathways involved in the interaction of TMBIM6 and miR-181a in breast cancer cells.

## Conclusion

The present study revealed that high expression of TMBIM6 in breast cancer is associated with cancer invasiveness. In addition, knockdown and overexpression of TMBIM6 modulated proliferation and migration of invasive breast cancer cells by regulating MAPK/ERK signaling pathway. Moreover, the expression of miR-181a was significantly affected by regulation of TMBIM6 expression. We therefore propose that TMBIM6-induced miR-181a upregulation could effectively regulate the migration and invasion of breast cancer.

## Supplementary Material

Figure S1: The box plot comparing specific Tmbim6 expression in normal and cancer tissue was derived from the ONCOMINE ™ database; Figure S2: Survival curve comparing patients with high and low expression in colon cancer, lung cancer, and gastric cancer was plotted from Kaplan Meier-plotter database.

## Figures and Tables

**Table 1:** List of primers used for quantification of specific gene expression.

Accession no.	Gene	Primer sequence	
NM_004360	*E-cadherin*	Forward	ACCACCTCCACAGCCACCGT
		Reverse	GTCCAGTTGGCACTCGCCCC
NM_001792	*N-cadherin*	Forward	TGTTTGACTATGAAGGCAGTGG
		Reverse	TCAGTCATCACCTCCACCAT
NM_003380	*Vimentin*	Forward	ACGCCATCAACACCGAGTTCA
		Reverse	GTGCCAGAGACGCATTGTCAA
NM_004994	*MMP-9*	Forward	TCTTCCAGTACCGAGAGAAAG
		Reverse	GGATGTCATAGGTCACGTAG
NM_005985	*Snail-1*	Forward	ACCACTATGCCGCGCTCTT
		Reverse	GGTCGTAGGGCTGCTGGAA
NM_003068	*Snail-2*	Forward	GGCAAGGCGTTTTCCAGAC
		Reverse	GCTCTGTTGCAGTGAGGGC
NM_005988	*ZEB-1*	Forward	CCCACCAAGTGCCAACCCCA
		Reverse	TGGACTGCAGGGCTGACCGT
NM_014795	*Sip-1*	Forward	GGAAGACAAGCTTCATATTGC
		Reverse	ATGGCTGTGTCACTGCGCTGA
NM_005438	*FOSL-1*	Forward	CTGTGCTTGAACCTGAGGCA
		Reverse	GGTGAAAGGAGTTAGGGAGGGT
NM_002228	*c-Jun*	Forward	AAGTAAGAGTGCGGGAGGCA
		Reverse	GGGCATCGTCATAGAAGGTCG
NM_002046	*GAPDH*	Forward	AGAACATCATCCCTGCATCC
		Reverse	CACCACCTTCTTGATGT

**Table 2:** List of primers used for quantification of specific miRNA gene expression.

miRNA		Primer sequence	
Tumor suppressor	*let-7a*	Forward	CTAGCCTGCAGGCAAGAAAGGTTAACATTAAATC
		Reverse	ATCCGGCCGGCCATTGAATTAGAGGCTTATAGCC
	*miR-19a*	Forward	CTAGCCTGCAGGCAGGTAGTGATGTGTGCATC
		Reverse	ATCCGGCCGGCCTGGATTTGCACAGCAGAATA
	*miR-125*	Forward	CTAGCCTGCAGGATAGGAGCTGGGGTGTCTTC
		Reverse	ATCCGGCCGGCCTGCCCACAAACAGCTGGCAGA
	*miR-133b*	Forward	ACACTCCAGCTGGGTTGGTCCCCTTCAACC
		Reverse	CTCAACTGGTGTCGTGGAGTCGGCAATTCAGTTGAGACAGCTGG
Oncogenic	*miR-17*	Forward	GAGCCAAAGTGCTTACAGTGC
		Reverse	AGTGCAGGGTCCGAGGTATT
	*miR-20*	Forward	TGGGTAAAGTGCTTATAGTGC
		Reverse	AGTGCAGGGTCCGAGGTATT
	*miR-155*	Forward	CTAGCCTGCAGGTATTCAAATATTTCCACAGA
		Reverse	ATCCGGCCGGCCTGAAGATGGTTATGAACATA
	*miR-181a*	Forward	CTAGCCTGCAGGCCTGCTTCTTTTCTTCTGTA
		Reverse	ATCCGGCCGGCCCTTTGGTTCTTCCTCCCACC
	*U6*	Forward	GCTTCGGCAGCACATATACTAAAAT
		Reverse	CGCTTCACGAATTTGCGTGTCAT

## Abbreviations

TMBIM: Transmembrane Bax Inhibitor Motif-containing
MMP: Matrix metalloproteinase
EMT: Epithelial to mesenchymal transition
ERK1/2: p42/p44 extracellular signal-related kinases
miRNA: MicroRNA
shRNA: small hairpin RNA
