# Supplementary material for: TMBIM6-mediated miR-181a expression regulates breast cancer cell migration and invasion via the MAPK/ERK signaling pathway
Source: J Cancer. 2023 Feb 22;14(4):554–72. doi: 10.7150/jca.81600 (PMC10088543; doi:10.7150/jca.81600)
Supplement: Supplementary file 1 — Figure S1: The box plot comparing specific Tmbim6 expression in normal and cancer tissue was derived from the ONCOMINE ™ database; Figure S2: Survival curve comparing patients with high and low expression in colon cancer, lung cancer, and gastric cancer was plotted from Kaplan Meier-plotter database. [file jcav14p0554s1.pdf]

Supplementary Figure legend

Figure S1. The box plot comparing specific TMBIM6 expression in normal (left plot) and cancer tissue (right plot) was derived from the ONCOMINE ™ database. (a) The TMBIM6 expression in superficial bladder cancer tissue compared to normal tissue from Sanchez-Carbayo Bladder 2 Statistics and Dyrskjot Bladder 3 Statistics, respectively. In addition, the comparison was analyzed in (b) lung adenocarcinoma from Okayama Lung Statistics, (c) activated B cell-like diffuse large B-cell lymphoma from Compagno Lymphoma Statistics, (d) colorectal adenoma from Skrzypczak Colorectal 2 Statistics, (e) diffuse gastric adenocarcinoma from Cho Gastric Statistics, (f) chronic lymphocytic leukemia from Haferlach Leukemia Statistics, and (g) B-cell childhood acute lymphoblastic leukemia from Haferlach Leukemia Statistics.

Figure S2. Survival curve comparing patients with high (red) and low (black, blue) expression in (a) colon cancer, (b) lung cancer, and (c) gastric cancer was plotted from Kaplan Meier-plotter database.

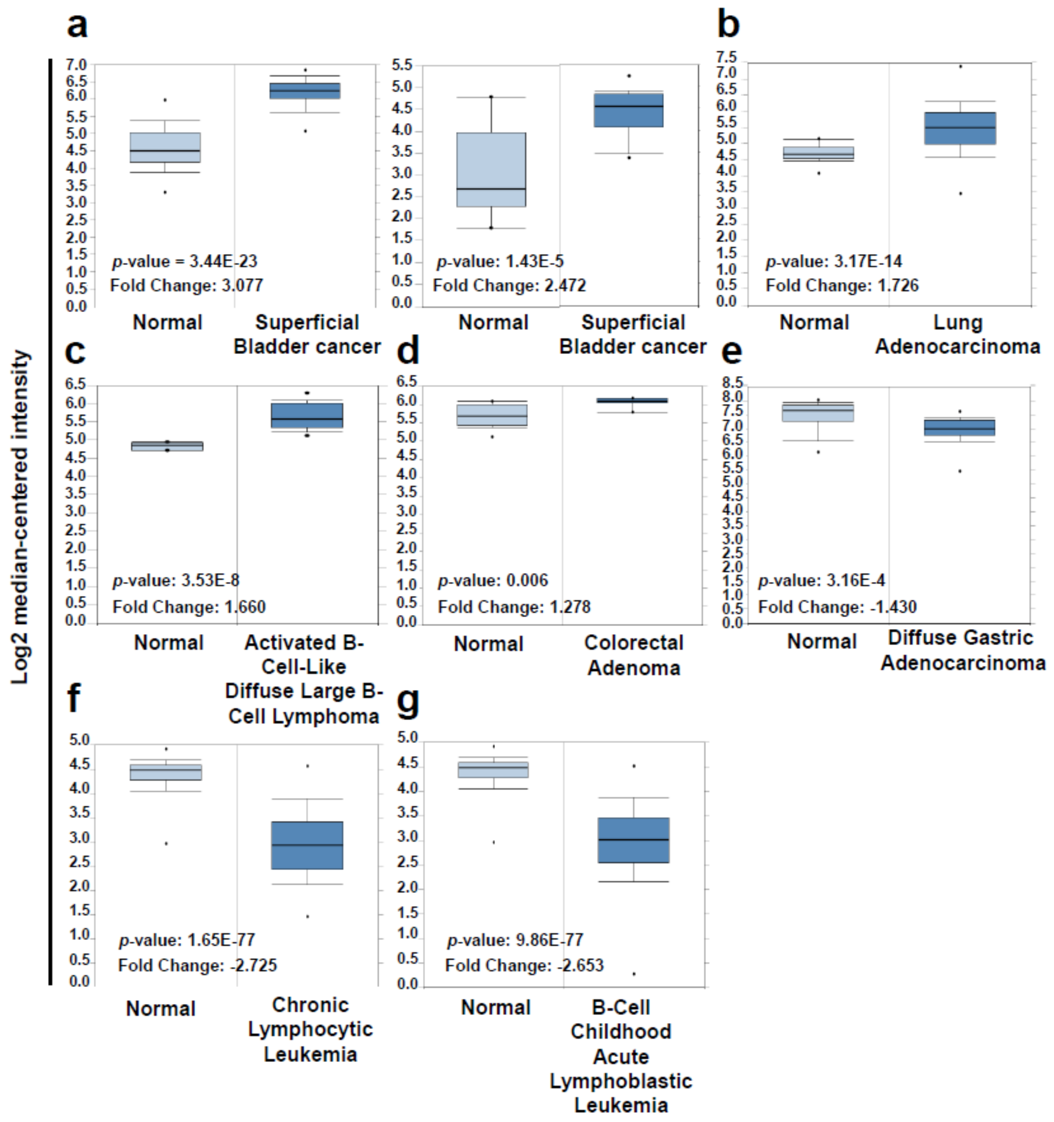

a
b
c
d
e
f
g
Log2 median-centered intensity
p-value = 3.44E-23
Fold Change: 3.077
Normal
Superficial Bladder cancer
p-value: 1.43E-5
Fold Change: 2.472
Normal
Superficial Bladder cancer
p-value: 3.17E-14
Fold Change: 1.726
Normal
Lung Adenocarcinoma
p-value: 3.53E-8
Fold Change: 1.660
Normal
Activated B-Cell-Like Diffuse Large B-Cell Lymphoma
p-value: 0.006
Fold Change: 1.278
Normal
Colorectal Adenoma
p-value: 3.16E-4
Fold Change: -1.430
Normal
Diffuse Gastric Adenocarcinoma
p-value: 1.65E-77
Fold Change: -2.725
Normal
Chronic Lymphocytic Leukemia
p-value: 9.86E-77
Fold Change: -2.653
Normal
B-Cell Childhood Acute Lymphoblastic Leukemia

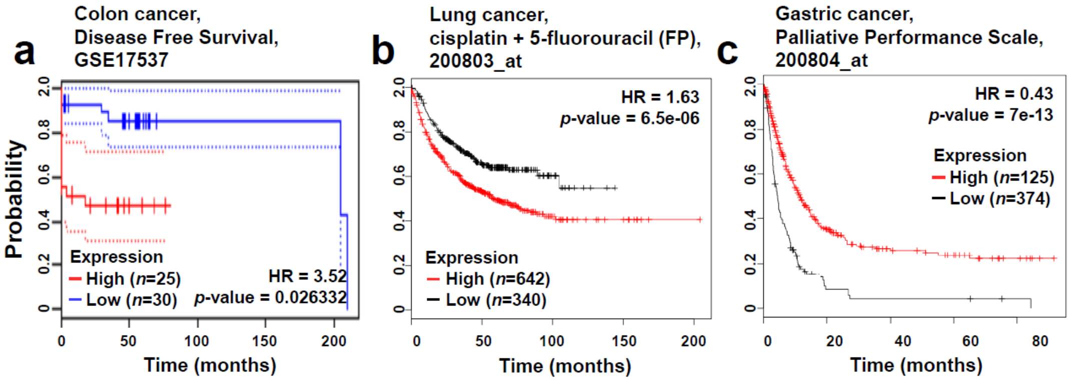
a
Colon cancer,
Disease Free Survival,
GSE17537
Probability
Expression
High (n=25)
Low (n=30)
HR = 3.52
p-value = 0.026332
Time (months)
b
Lung cancer,
cisplatin + 5-fluorouracil (FP),
200803_at
HR = 1.63
p-value = 6.5e-06
Expression
High (n=642)
Low (n=340)
Time (months)
c
Gastric cancer,
Palliative Performance Scale,
200804_at
HR = 0.43
p-value = 7e-13
Expression
High (n=125)
Low (n=374)
Time (months)
